# Free electrons spin-dependent Kapitza–Dirac effect in two-dimensional triangular optical lattice

**DOI:** 10.1515/nanoph-2024-0191

**Published:** 2024-07-12

**Authors:** Jiahao Tian, Fang Liu, Xiaotong Xiong, Yidong Huang

**Affiliations:** Department of Electronic Engineering, 12442 Tsinghua University, Beijing 100084, China; Beijing National Research Center for Information Science and Technology, 12442 Tsinghua University, Beijing 100084, China

**Keywords:** free electron, spin-dependent Kapitza-Dirac effect, triangular optical lattice, spatial inversion symmetry breaking

## Abstract

The free electron spin dynamics in Kapitza–Dirac (KD) effect had been studied theoretically in one-dimensional standing wave of EUV to X-ray laser with extremely high intensity, which is far beyond experimental realization. Here, we propose to achieve the free electron spin-dependent KD effect in two-dimensional triangular optical lattice with spatial inversion symmetry breaking, and the theoretical results reveal that laser with wavelength in visible or near-IR and five orders of magnitude decreased intensity could lead to obvious spin-dependent KD effect. This work provides the way to realize the free electron spin-dependent KD effect experimentally.

## 1 Introduction

The interaction between light and free electrons has received widespread attention. Different from the conventional free electron radiation and free electron acceleration by light, the quantum properties of free electrons have been gradually revealed in near-field optics and in free space. As interacting with the optical near-field, the free electron wavefunction [1], [2], [3], [4], [5], [6] and photon states [7], [8], [9] can be manipulated and shaped based on the photon-induced near-field electron microscopy (PINEM) effect [10], [11]. While interacting with the photons in free space, the free electrons can be scattered elastically or inelastically by photons which is known as Compton scattering and gives rise to research on Compton imaging [12], [13], high-energy photon emission source [14], [15], [16], and so on. Stimulated Compton scattering has also been studied due to the enhanced interaction strength of electrons and photons in free space [17], [18], [19], [20].

As a kind of stimulated Compton scattering, the Kapitza–Dirac (KD) effect shows transverse momentum modulation of free electron by absorbing and emitting a photon with opposite momentum at the same time in a standing wave [21], [22], [23], [24], [25], which is essentially the diffraction of electrons by the periodic optical potential macroscopically. Shortly after the first experimental realization of the KD effect, the two-color KD effect was predicted, corresponding to the higher order quantum scattering process between electrons and photons in free space [26], [27]. The progress in ultrafast laser technology makes the research on ultrafast dynamics possible in KD effect and some new phenomena has been detected in the ultrafast KD effect [28], prompting explorations on ultrafast dynamics between free electrons and photons. Furthermore, the important role of the interference between quantum paths was also highlighted [29]. It opens the door to the study of the KD effect in arbitrary light fields such as structured light, and paves the way to realize the interaction between high-dimensional optical modes and free electrons [30], [31].

In the spin-dependent KD effect, the spin of electrons undergoes periodic precession in one-dimensional standing wave formed by circularly polarized light [32], [33], [34]. It shows an extra-dimension of the interaction between the electrons and the electromagnetic field, and provides a platform to study the spin-dependent scattering process and coherent control the spin electrons states [35], [36]. However, spin-dependent KD effect had not been observed experimentally. The theoretical results indicate that, in order to make the spin effect significant, the wavelength of the standing wave should be shortened to the extreme ultraviolet (EUV) or even hard X-ray, and the corresponding laser intensity needs to be increased to 10^19^ ∼ 10^22^ W/cm^2^ [32], [33], [34], [35], [36]. These extreme conditions could not be reached based on the existing laser system even if the complicated optical system in EUV and X-ray might be tolerated.

Here we propose a scheme to achieve the spin-dependent KD effect by having free electrons interact with a 2D triangular optical lattice, which is formed by the laser with wavelength in visible or near-IR and greatly decreased intensity instead of EUV or X-ray laser with extreme high intensity. It is found theoretically that, by adjusting the polarization angle *θ* of the linearly polarized laser beams, the weakened spin-independent potential and relatively enhanced spin-dependent potential in 2D optical lattice could greatly relax the conditions required for the spin-dependent KD effect. The numerical solutions explicitly give diffraction patterns of electrons showing obvious spatial inversion symmetry breaking, which results from the spatial inversion symmetry breaking ponderomotive potential. Electrons carrying opposite spin produce complementary diffraction patterns, indicating spin-dependent KD effect.

## 2 Theoretical scheme

**Figure 1:**
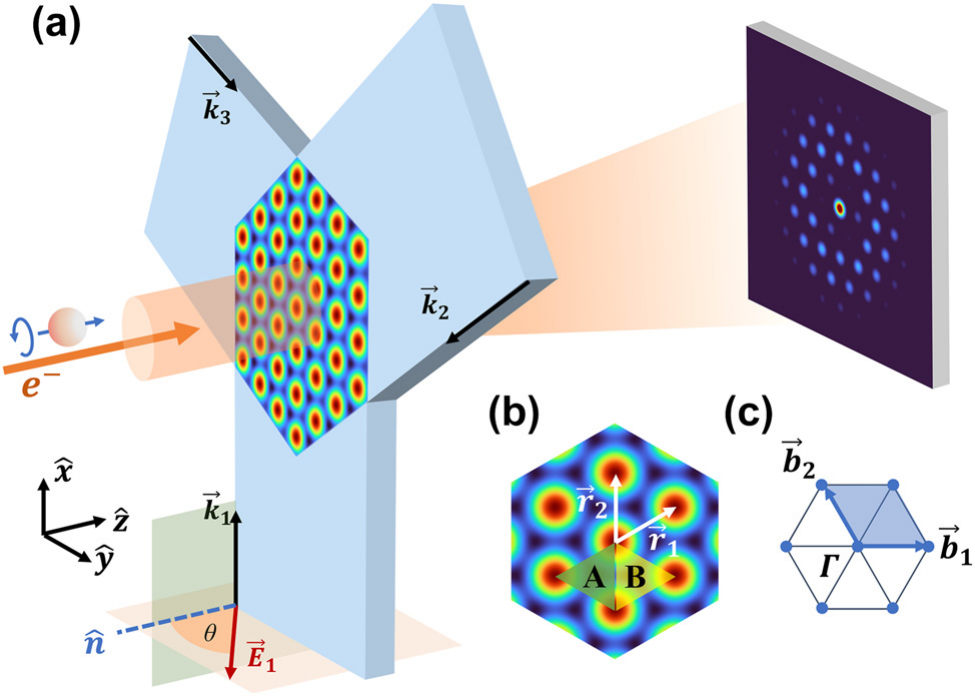
Schematic diagram of free electrons spin-dependent Kapitza–Dirac (KD) effect in two-dimensional (2D) triangular optical lattice. (a) Three linearly polarized lasers are incident in the plane at 120° with respect to each other, which form a periodic intensity distribution, namely an optical lattice. The polarization directions of the three lasers are all rotated from $\hat{n}$ to ${\hat{k}}_{i}\times \hat{n}$ by *θ*. The electrons with the spin direction of $+\hat{z}$ or $-\hat{z}$ are incident along *z* direction and diffract into pattern with broken spatial inversion symmetry. (b) Spatial distribution of the potential experienced by electrons, which is proportional to the intensity of the optical lattice. ${\vec{r}}_{1}$ and ${\vec{r}}_{2}$ are the lattice vectors, while *A* and *B* are different sites in the optical lattice. The length of the lattice vector is $\left|{\vec{r}}_{1}\right|=\left|{\vec{r}}_{2}\right|=2/3\lambda$ . (c) The *k*-space of the lattice. ${\vec{b}}_{1}$ and ${\vec{b}}_{2}$ are the reciprocal lattice basis vectors. Γ is the origin in reciprocal space, corresponding to the zero-order diffraction spot.

The triangular 2D optical lattice is constructed by three linearly polarized visible or near-IR laser beams with the same wavelength and intensity, as shown in Figure 1(a). Three laser beams intersect in *x*–*y* plane at an angle of 120° with each other, forming a spatial periodic intensity distribution as well as a periodic ponderomotive potential for electrons by interference. The free electrons inject normally into the optical lattice (along *z*-axis) and the KD effect would take place in Raman–Nath regime. The following calculation indicates that the ponderomotive potential of 2D triangular optical lattice is electron spin-dependent and spatial inversion symmetry breaking, which results in the electron diffraction pattern with broken spatial inversion symmetry. Free electrons with opposite spin directions exhibit complementary diffraction patterns, so that the spins of the electrons incident into the optical lattice can be distinguished by the diffraction patterns.

The angle between the polarized laser beam and the normal direction of the optical lattice (*z* axis) is defined as *θ* shown in Figure 1(a). And as shown in Figure 1(b) and (c), we define ${\vec{r}}_{1}$ , ${\vec{r}}_{2}$ as the lattice vectors analogous to those in atomic lattice and ${\vec{b}}_{1}$ , ${\vec{b}}_{2}$ as the corresponding reciprocal lattice basis vectors with ${\vec{r}}_{i}{\vec{b}}_{j}=2\pi \delta_{ij}$ . According to the laser polarization, the optical lattice can be divided into two types. In the case of *θ* = *π*/2, the polarization of laser beam is parallel to the optical lattice plane (*x*–*y* plane). The electric field $\vec{E}$ at the center of site *A* and *B* can be written as Eq. (1a), which indicates that there exists phase difference between *E*_*x*_ and *E*_*y*_ and the electric field rotates in the plane of the optical lattice. In the case of *θ* = 0, the polarization of laser beam is perpendicular to the optical lattice plane and the magnetic field $\vec{B}$ is parallel to the optical lattice plane. Equation (1b) indicates the magnetic field rotates in *x*–*y* plane, similar to the electric field when *θ* = *π*/2. Any other polarizations could be divided into the above two cases. Thus, this 2D lattice has the property of field rotation in *x*–*y* plane, which is due to the interference of three linearly-polarized laser beams rather than the spin of the laser beam. This field rotation is defined here as the pseudospin of the optical lattice. Besides, Eq. (1) indicates that the pseudospin of the optical lattice is site-dependent and such an optical lattice shows spatial inversion symmetry breaking.

(1a) $$\begin{array}{c} {\vec{E}}_{A/B,\theta =\pi /2}=\frac{3}{2}\left|E\right|{\text{e}}^{-\text{i}\omega t}\left(\hat{x}+{\text{e}}^{\pm \frac{\text{i}\pi }{2}}\hat{y}\right) \end{array}$$

(1b) $${\vec{B}}_{A/B,\theta =0}=\frac{3}{2}\left|B\right|{\text{e}}^{-\text{i}\omega t}\left(\hat{x}+{\text{e}}^{\pm \frac{\text{i}\pi }{2}}\hat{y}\right)$$

Unlike the previous work where the electron beam is incident into the standing wave at the Bragg angle [33], [34], [35], in this paper the incident direction of electron beam is perpendicular to the plane of the optical lattice, and the KD effect is in the Raman–Nath regime. To separate the matter and anti-matter components, the weak relativistic Dirac equation is introduced by using the Foldy–Wouthuysen transformation [37],

(2) $$\begin{array}{c} i\hbar \frac{\partial }{\partial t}\psi =\left(\frac{{\left[-i\hbar \nabla -q\vec{A}\right]}^{2}}{2m}-\frac{q\hbar }{2m}\vec{\sigma }\cdot \vec{B}-\frac{{\left[-i\hbar \nabla -q\vec{A}\right]}^{4}}{8m^{3}c^{2}}\right.\\ \;-\left.\frac{q\hbar }{4m^{2}c^{2}}\vec{\sigma }\cdot \left\{\vec{E}\times \left(-i\hbar \nabla -q\vec{A}\right)\right\}\right.\\ \left.+\frac{q\hbar }{8m^{3}c^{2}}\left\{\vec{\sigma }\cdot \vec{B},{\left[-i\hbar \nabla -q\vec{A}\right]}^{2}\right\}\right)\psi \end{array}$$

where *ψ* is Pauli spinor, *m* is electron mass, *q* is electron charge, $\vec{A}$ is the vector potential and $\vec{\sigma }=\left(\sigma_{x},\sigma_{y},\sigma_{z}\right)$ is the Pauli matrices. The first term and the second term on the right side are consistent with the Schrödinger equation, while the third term is the relativistic correction to the first term. The fourth term on the right side leads to the spin–orbit coupling and coupling of the optical lattice pseudospin to the electron spin. The last term is anticommutator, representing the correction to the Zeeman coupling [33]. In the triangular optical lattice, the vector potential ${\vec{A}}_{i}$ of three lasers with angular frequency *ω* is

(3) $${\vec{A}}_{i}=\frac{{\vec{E}}_{i}}{\omega }\sin\left({\vec{k}}_{i}\cdot \vec{r}-\omega t\right),i=1,2,3$$

where ${\vec{k}}_{i}$ is the wave vector of three laser beams. To derive the time-independent potential function of free electrons in the optical lattice, we substitute Eqs. (3) into (2) and utilize Magnus expansion to the second order [38], [39]. Ignoring the items in the Magnus expansion that do not grow linearly with time and the high-order small quantities in the visible to near-infrared band, the wave equation can be written as follows,

(4a) $$i\hbar \frac{\partial }{\partial t}\psi =\left(-\frac{\hbar^{2}}{2m}\nabla^{2}+V_{0}+V_{\text{spin}}+V_{\text{p}}\right)\psi$$

(4b) $$V_{0}=\Omega_{0}\left(\frac{mc^{2}}{2\hbar \omega }\right)\left(-\frac{1}{2}\sin^{2}\left(\theta \right)+\cos^{2}\left(\theta \right)\right)\underset{i}{\sum }\cos\left({\vec{Q}}_{i}\cdot \vec{r}\right)$$

(4c) $$V_{\text{spin}}=\frac{\sqrt{3}}{8}\Omega_{0}\vec{\sigma }\cdot \hat{z}\left(\sin^{2}\left(\theta \right)-\cos^{2}\left(\theta \right)\right)\underset{i}{\sum }\sin\left({\vec{Q}}_{i}\cdot \vec{r}\right)$$

(4d) $$V_{\text{p}}=\frac{\sqrt{3}}{4}\Omega_{0}\vec{\sigma }\cdot \sin\left(2\theta \right)\underset{i}{\sum }{\hat{Q}}_{i}\sin\left({\vec{Q}}_{i}\cdot \vec{r}\right)$$

where $\Omega_{0}=\frac{{\left(qE_{0}\hbar c\right)}^{2}}{\hbar \omega {\left(mc^{2}\right)}^{2}}$ represents the general scale of ponderomotive potential, *V*_0_ is the spin-independent ponderomotive potential of the optical lattice for electrons, *V*_spin_ and *V*_*p*_ are the spin-dependent ponderomotive potentials, corresponding to the inversion asymmetric ponderomotive force and the spin precession, respectively. *E*_0_ is the electric field amplitude. ${\vec{Q}}_{1}={\vec{b}}_{1}$ , ${\vec{Q}}_{2}={\vec{b}}_{2}$ and ${\vec{Q}}_{3}=-\left({\vec{b}}_{1}+{\vec{b}}_{2}\right)$ are introduced for compactness. The relation between ${\vec{Q}}_{i}$ and wave vectors ${\vec{k}}_{i}$ are ${\vec{k}}_{i}-{\vec{k}}_{j}={\vec{Q}}_{l}$ , where (*i*, *j*, *l*) ∈ {(3,2,1), (1,3,2), (2,1,3)}, and ${\hat{Q}}_{i}$ is the direction vector of ${\vec{Q}}_{i}$ which fulfills $\sum_{i}{\vec{Q}}_{i}=0$ . To keep the KD effect in Raman–Nath regime, the total ponderomotive energy should be higher than the electrons recoil kinetic energy [23].

**Figure 2:**
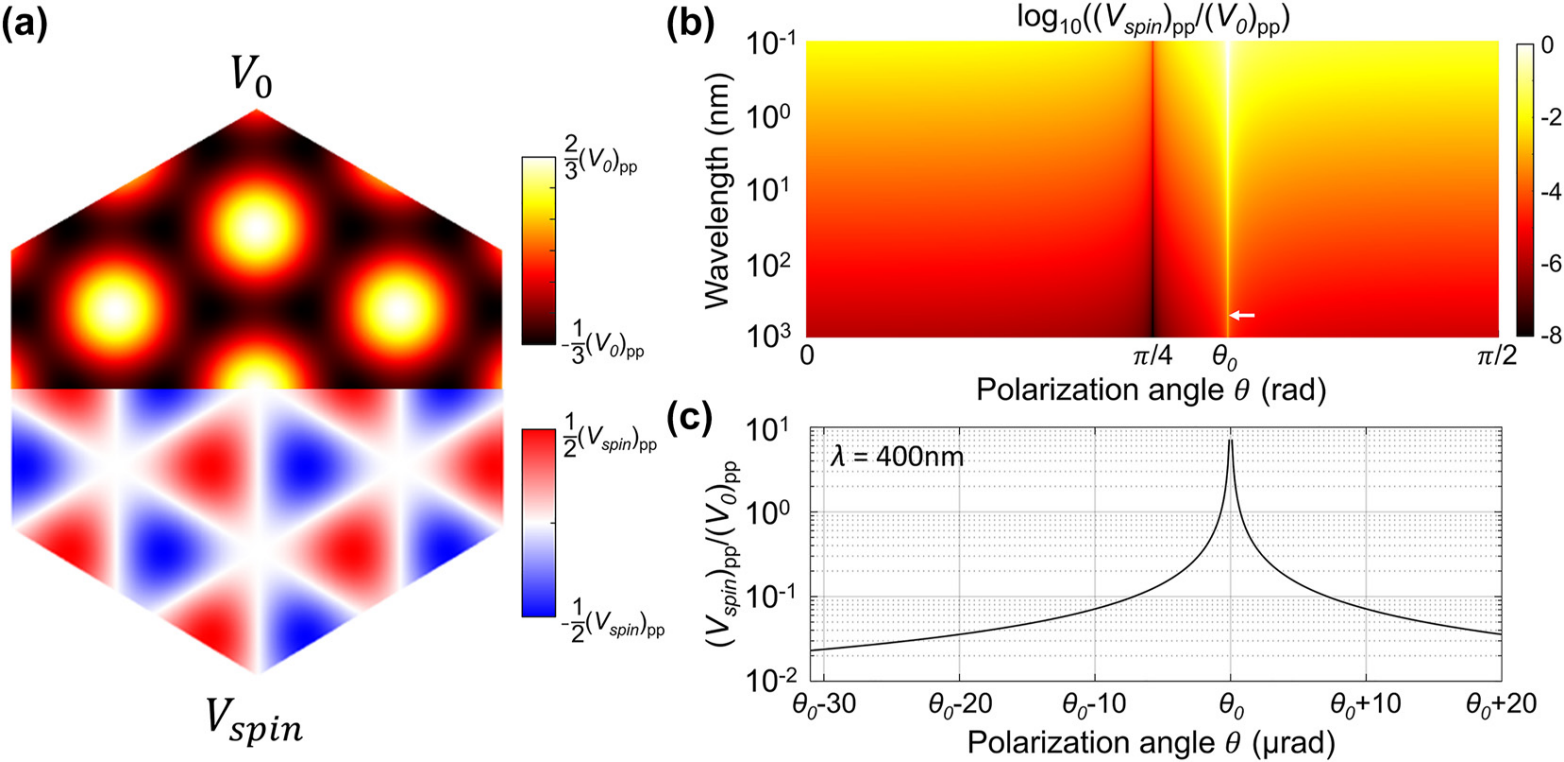
Spatial inversion symmetry properties of ponderomotive potentials and the relation between the amplitude ratio ${\left(V_{\text{spin}}\right)}_{pp}/{\left(V_{0}\right)}_{pp}$ and wavelength and polarization angle. (a) The upper and lower parts are *V*_0_ and *V*_spin_, respectively, indicating *V*_0_ is spatial inversion symmetric while *V*_spin_ is not. (b) ${\left(V_{\text{spin}}\right)}_{pp}/{\left(V_{0}\right)}_{pp}$ as a function of wavelength and polarization angle. For wavelength *λ* = 0.1 nm, ${\left(V_{\text{spin}}\right)}_{pp}/{\left(V_{0}\right)}_{pp}$ is 4 orders of magnitude higher than that in the visible or near-IR band. The white arrow shows the enhancement to the order of 10^−2^ in the vicinity of *θ*_0_ when *λ* = 400 nm, and the detail is plotted in (c).

The spin-dependent ponderomotive potentials originate from the pseudospin of optical lattice, which leads to non-vanishing time-independent term in $\vec{\sigma }\cdot \left(\vec{E}\times q\vec{A}\right)$ and in Magnus expansion of $\vec{\sigma }\cdot \vec{B}$ . Considering that the electron spin direction is perpendicular to the optical lattice plane (*x*–*y* plane), the electrons will carry additional spin-dependent energy by *V*_spin_ according to Eq. (4c), which contains only the *z*-component Pauli matrix *σ*_*z*_. As a result, the electrons experience additional ponderomotive force because of *V*_spin_. On the other side *V*_*p*_ corresponds to the electron spin precession only. For spin-up electrons, *V*_0_ and *V*_spin_ are illustrated as the upper half and lower half of Figure 2(a), which are actually decided by the spatial modulation function $\sum_{i}\cos\left({\vec{Q}}_{i}\cdot \vec{r}\right)$ and $\sum_{i}\sin\left({\vec{Q}}_{i}\cdot \vec{r}\right)$ in Eq. (4), respectively. It can be seen that *V*_0_ is spatial inversion symmetric, which would result in the spatial inversion symmetric diffraction pattern of electrons. *V*_spin_ shows spatial inversion symmetry breaking, leading to the electron diffraction pattern with broken spatial inversion symmetry. Thus, the amplitude ratio of potentials *V*_spin_ and *V*_0_ is defined as

(5) $$\frac{{\left(V_{\text{spin}}\right)}_{pp}}{{\left(V_{0}\right)}_{pp}}=\left|\left(\frac{\hbar \omega }{2mc^{2}}\right)\frac{\cos\left(2\theta \right)}{1-3/2\sin^{2}\left(\theta \right)}\right|$$

where ${\left(V_{\text{spin}}\right)}_{pp}$ and ${\left(V_{0}\right)}_{pp}$ denotes the peak-to-peak value of *V*_spin_ and *V*_0_, respectively, for revealing whether the feature of spatial inversion symmetry breaking of electron diffraction pattern is obvious or not.

With the potential, the spin-dependent electron diffraction patterns can be further obtained by calculating the wave function $\left|\psi \right|$ in Eq. (4a). In our calculations, we assume that the electron is an ideal plane wave vertically incident into the light lattice with no transverse momentum, that is, the initial state wave function is $\psi \left(0\right)=c\left(0\right){\text{e}}^{\text{i}\vec{p}\cdot \vec{r}}$ where $\vec{p}$ is the initial momentum and $c\left(0\right)$ is the initial spin state of the free electron. The optical lattice extends to infinity in the *x*–*y* plane and has ideal periodic translation symmetry. In such an optical lattice, the transverse momentum of electrons is modulated. Since the terms linearly to field components is ignored, the initial momentum of the electron and the thickness of the optical lattice in electron traversing direction determine the interaction time and thereby affect the interaction between the electron and the optical lattice. For the same reason the initial momentum component ${\text{e}}^{\text{i}\vec{p}\cdot \vec{r}}$ in the electron wave function is ignored below. According to energy and momentum conservation, the transverse momentum distribution of electrons after passing through the optical lattice is $m{\vec{b}}_{1}+n{\vec{b}}_{2},m,n\in Z$ . Therefore, the wave function of electrons can be expressed as $\psi \left(t\right)=\sum_{\text{m},\text{n}\in Z}c_{m,n}\left(t\right){\text{e}}^{\text{i}\left(\text{m}{\vec{b}}_{1}+\text{n}{\vec{b}}_{2}\right)\cdot \vec{r}}$ , where $c_{m,n}\left(t\right)=\left[c_{m,n}^{↑}\left(t\right),c_{m,n}^{↓}\left(t\right)\right]$ is a Pauli spinor containing the wave function expansion coefficients of electrons with opposite spin directions. Substituting the wave function into Eq. (4a) and setting the initial condition $c\left(0\right)=c_{0,0}\left(0\right)=\left[1,0\right]$ or $\left[0,1\right]$ for the spin $\pm \hat{z}$ , the coefficients $c_{m,n}\left(t\right)$ and the diffraction probability $P_{m,n}\left(t\right)={\left|c_{m,n}\left(t\right)\right|}^{2}$ , as well as the electron diffraction pattern, could be calculated.

## 3 Spin-dependent electron diffraction results

### 3.1 The selection of polarization angle in 2D optical lattice

In order to obtain spin-dependent diffraction effect obviously, two conditions should be satisfied simultaneously. One is the Raman–Nath condition, that is, ${\left(V_{\text{spin}}+V_{0}\right)}_{pp}$ should be larger than the electron recoil kinetic energy. Another is that ${\left(V_{\text{spin}}\right)}_{pp}/{\left(V_{0}\right)}_{pp}$ must be relatively large. The following discussions are about how to set the laser intensity, wavelength *λ* and polarization angle *θ* of 2D triangular optical lattice to meet the above two conditions.

For the strong laser intensity excited optical lattice, according to Eqs. (4b) and (4c), ${\left(V_{\text{spin}}+V_{0}\right)}_{pp}$ is very large (see Figure S1 in the Supplementary Materials [40]) and the Raman–Nath condition is easily satisfied. Further considering ${\left(V_{\text{spin}}\right)}_{pp}/{\left(V_{0}\right)}_{pp}$ according to Eq. (5), its value is plotted in Figure 2(b) as functions of *λ* and *θ*. For short wavelength, e.g. *λ* = 0.1 nm, ${\left(V_{\text{spin}}\right)}_{pp}/{\left(V_{0}\right)}_{pp}$ is relatively large (approximately 10^−2^) for most of *θ*. While, for long wavelength, e.g. *λ* = 400 nm, *θ* should be confined in the vicinity of *θ*_0_ ( $\arcsin\left(\sqrt{2/3}\right)$ ) to get a high ${\left(V_{\text{spin}}\right)}_{pp}/{\left(V_{0}\right)}_{pp}$ value. For example, as shown in Figure 2(c), ${\left(V_{\text{spin}}\right)}_{pp}/{\left(V_{0}\right)}_{pp}$ is larger than ∼0.03 when $\left|\Delta \theta \right|=\left|\theta -\theta_{0}\right|\leq 20\;\mu \text{rad}$ , which has been increased by nearly four orders of magnitude compared with those when *θ* = 0, *π*/2. Thus, for 2D triangular optical lattice excited by strong laser in the visible or near-IR band, we only need to make Δ*θ* small so that ${\left(V_{\text{spin}}\right)}_{pp}/{\left(V_{0}\right)}_{pp}$ is large enough.

For the weak laser intensity excited optical lattice, ${\left(V_{\text{spin}}+V_{0}\right)}_{pp}$ becomes very small as the *V*_0_ vanishes at *θ*_0_ (see Figure S1 in the Supplementary Materials [40]), so that *θ* should not be too close to *θ*_0_ to meet the Raman–Nath condition. Besides, ${\left(V_{\text{spin}}\right)}_{pp}/{\left(V_{0}\right)}_{pp}$ should be further considered according to Figure 2(b). For short wavelength, ${\left(V_{\text{spin}}\right)}_{pp}/{\left(V_{0}\right)}_{pp}$ is relatively large (approximately 10^−2^) for most of *θ*. While, for long wavelength, *θ* should also be confined in the vicinity of *θ*_0_ (micro-radians level) to obtain relatively large ${\left(V_{\text{spin}}\right)}_{pp}/{\left(V_{0}\right)}_{pp}∼10^{-2}$ , which is improved by 4 orders of magnitude. Therefore, to obtain significant spin-dependent diffraction under weak laser intensity, Δ*θ* should be small to have relatively large ${\left(V_{\text{spin}}\right)}_{pp}/{\left(V_{0}\right)}_{pp}$ but should not be too close to zero to satisfy the Raman–Nath condition.

In all, to obtain spin-dependent electron diffraction by visible or near-IR 2D triangular optical lattice, the laser intensity and *θ* should be considered simultaneously as follows. (1) For strong laser intensity, *θ* should be in the vicinity of *θ*_0_ (tens of micro-radians level); (2) For weak laser intensity, *θ* should be in the vicinity of but not too close to *θ*_0_.

### 3.2 Spin-dependent diffraction patterns with 2D optical lattice

**Figure 3:**
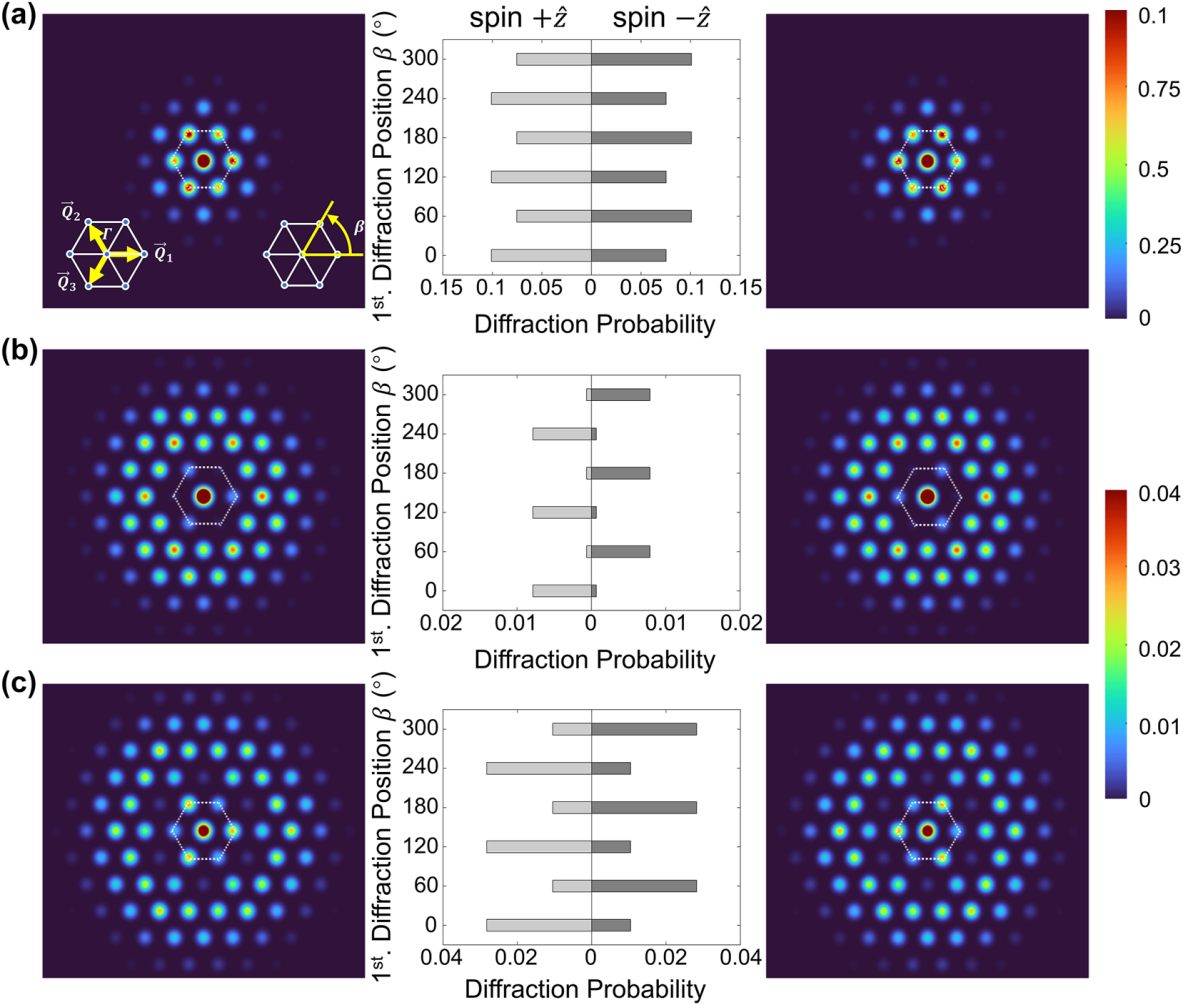
Diffraction patterns and probability of free electrons with the interaction time *τ* = (a) 3,000*T*, (b) 6,253*T,* and (c) 7,500*T* (*T* represents the laser oscillation period). The left and the right panels are the 1st to 5th order diffraction pattern with spin direction $+\hat{z}$ and $-\hat{z}$ , respectively. Each spot in the pattern corresponds to a certain reciprocal point of the optical lattice. The white dashed line in each figure indicates where the 1st order diffraction pattern locates, which corresponds to the left inset of (a). In the middle panels, the diffraction probability of the six 1st-order spots with $+\hat{z}$ (left half) and $-\hat{z}$ (right half) spin varies with the spots location identified by the degree, which is illustrated as the right inset of (a). The diffraction probabilities are extracted from the left and the right panels, respectively. Here, the optical lattice is formed by laser beams with *λ* = 400 nm and *E* = 5.0 × 10^10^ **V/m (intensity *I* = 3.32 × 10^14^ W/cm^2^).

According to the theoretical analysis, the spin-dependent electron diffraction pattern can be numerically calculated under specific conditions. At the polarization angle *θ* = 54.735° (Δ*θ* ≈ −11 μrad), for optical lattice with laser wavelength *λ* = 400 nm and electric field amplitude *E* = 5.0 × 10^10^ V/m (corresponding to *I* = 3.32 × 10^14^ W/cm^2^), Figure 3 illustrates the calculated electrons diffraction pattern exhibiting obvious spin-dependent KD effect. The left and the right panels show the diffraction pattern of electrons with $+\hat{z}$ and $-\hat{z}$ spin, respectively. The final diffraction pattern is directly influenced by the duration time of free electrons in the optical lattice, which is defined as the interaction time *τ*. The momentum distribution of electrons changes continuously while interacting with the optical lattice, leading to different diffraction patterns with different *τ*. Figure 3 illustrates the diffraction pattern with *τ =* 3,000, 6,253, and 7,500 *T* (*T* represents the laser oscillation period, and *τ* ≈ 4.0 ps, 8.3 ps, and 10.0 ps), respectively. In the electron diffraction pattern, we can find the principal component (the central diffraction spot), the six 1st-order diffraction spots around the central diffraction spot, and the 2nd-order to 5th-order diffraction patterns in the outer ring of the 1st-order diffraction pattern.

The 1st-order diffraction spots show the property of spatial inversion symmetry breaking, especially in Figure 3(b) and (c). The three obvious spots, as well as the three inconspicuous spots, reveal a C_3_ symmetry rather than C_6_ symmetry around the electron beam axis. In order to illustrate the spatial inversion symmetry breaking of diffraction pattern more obviously and quantitatively, the middle panels in Figure 3 draw the spin-dependent diffraction probability of the six first-order spots, whose locations are described by degree *β* = 0, 60°, …, 300°. Left and right half correspond to spin direction $+\hat{z}$ and $-\hat{z}$ , which illustrate that there exists significant difference of diffraction probability for the same spot *β*. This is consistent with the nature of the optical lattice with broken spatial inversion symmetry.

For the second to fourth order diffraction pattern, the C_3_ symmetry feature can still be identified in spite of the lower diffraction probability (see the diffraction pattern and probabilities at higher-order spots in Supplementary Materials [40]). Therefore, the calculation results in Figure 3 reveal the spin-dependent KD effect and confirm that the relatively enhanced *V*_spin_ could lead to the KD effect. Theoretically, if the laser intensity of the optical lattice is enhanced, the electrons would be scattered to diffraction spots of higher order (beyond the ±5th order) and the spin-dependent diffraction pattern could be distinguished there.

### 3.3 The visibility of spin-dependent diffraction pattern and the discussions

**Figure 4:**
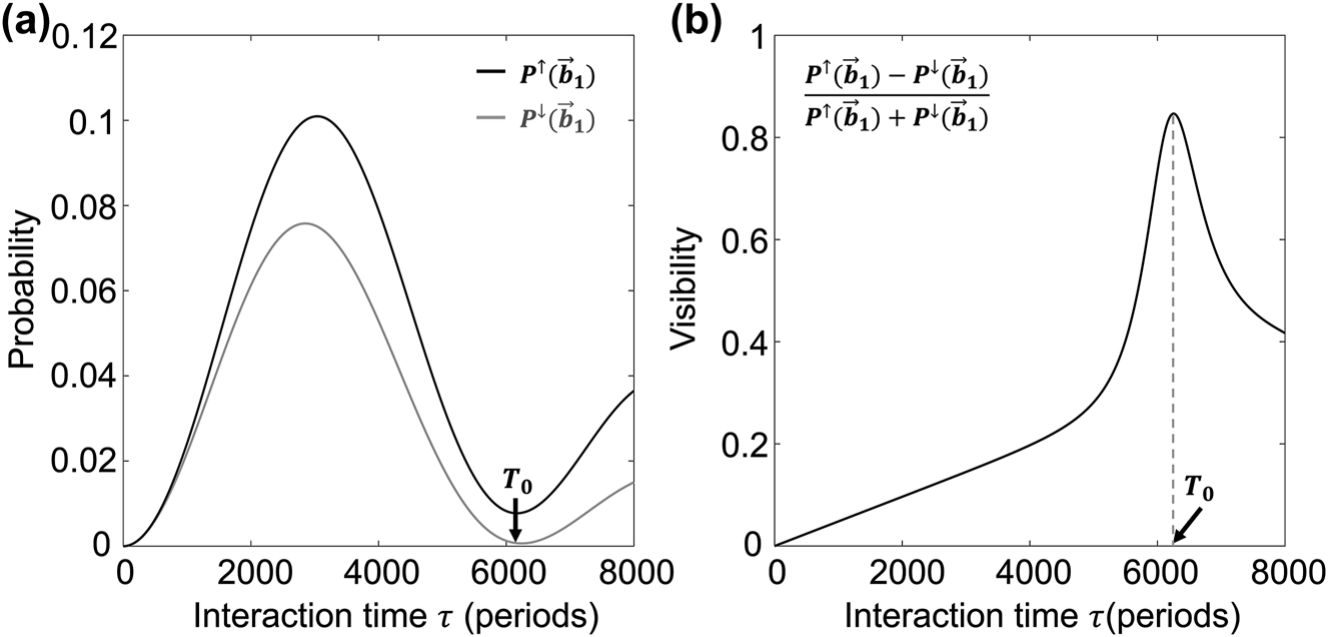
The evolution of the diffraction probabilities and the visibility. (a) The probabilities at the spot ${\vec{b}}_{1}$ . *P* ^↑^ and *P* ^↓^ correspond to electrons carrying spin $+\hat{z}$ and $-\hat{z}$ . (b) The visibility of ${\vec{b}}_{1}$ diffraction spots of opposite spin versus interaction time. Plotted within the first 8,000*T* (*τ* ≈ 10.7 ps).

Considering that the 1st-order diffraction pattern has less diffraction states and obvious different diffraction pattern, it is more reasonable to verify the spin effect in the 1st-order diffraction pattern. Figure 4(a) illustrates the calculated electron diffraction probabilities of spin $\pm \hat{z}$ , $P^{↑}\left({\vec{b}}_{1}\right)$ and $P^{↓}\left({\vec{b}}_{1}\right)$ , as a function of interaction time ( ${\vec{b}}_{1}$ is one of the 1st-order electron diffraction spots as shown in the left inset of Figure 3(a)). It can be seen that the $P^{↑}\left({\vec{b}}_{1}\right)$ and $P^{↓}\left({\vec{b}}_{1}\right)$ oscillate when extending interaction time, corresponding to the shift of electrons among the central, the 1st-order and the 2nd-order diffraction states, and the probability difference between the opposite spins can be verified clearly. In order to quantify electrons spin effect, the visibility, $V=\frac{P^{↑}\left({\vec{b}}_{1}\right)-P^{↓}\left({\vec{b}}_{1}\right)}{P^{↑}\left({\vec{b}}_{1}\right)+P^{↓}\left({\vec{b}}_{1}\right)}\in \left[-1,1\right]$ , is introduced here to describe the normalized probability difference of opposite spin $\pm \hat{z}$ in the spots of ${\vec{b}}_{1}$ as depicted in Figure 4(b). The visibility of the 1st-order diffraction pattern increases gradually and then reaches a local maximum, but the diffraction probabilities, $P^{↑}\left({\vec{b}}_{1}\right)$ and $P^{↓}\left({\vec{b}}_{1}\right)$ , reach a local minimum at *T*_0_. The corresponding diffraction pattern when the visibility reaches local maximum is depicted in Figure 3(b), indicating that the property of spatial inversion symmetry breaking around *T*_0_ is more obvious compared to those in Figure 3(a) and (c). In the one-dimensional KD effect in Raman–Nath regime, the probabilities evolution of each order diffraction fit the square of the first-kind Bessel functions very well. This relation in two-dimensional KD effect, however, is only roughly approximated, with no zero-crossing moment. So the moment when the first order diffraction probabilities reach their first minimum could only be estimated roughly as $T_{0}∼j_{1,1}\hbar /\left(3{\left(V_{0}\right)}_{pp}\right)$ , where *j*_1,1_ is the first root of the first-order Bessel function, ${\left(V_{0}\right)}_{pp}$ is the peak-peak amplitude of *V*_0_, and the factor 3 results from the triples of electron diffraction paths in triangular optical lattice compared to those in one-dimensional standing wave. This *T*_0_ for estimating the maximum visibility also works well for different situations as shown in Figures S3 and S4. It thus provides an instruction to determine the electron propagation length with specific electron kinetic energy for capturing diffraction pattern with obvious broken spatial inversion symmetry.

As stated above and shown in Table 1, the spin effect in the 2D optical lattice can be obtained with greatly relaxed conditions. This is mainly because the physical mechanisms of spin-dependent KD induced by 1D standing wave and 2D optical lattice are quite different. In 1D standing wave, the electron spin effect reflects in periodic spin flipping because of the spin precession during the electrons interacting with the standing wave [32], [33], [34], [35], [36]. Different from previous works, in 2D optical lattice, the spin coupling comes from the pseudospin of the optical field, rather than the spin of the photon itself. The electron spin effect reflects in the diffraction pattern and the visibility, which shows the spatial inversion symmetry breaking. By adjusting the polarization of the laser, the spin effect of electron diffraction can be produced in the optical lattice, which is created by the visible to near-infrared lasers. By the way, the potential *V*_*p*_ in Eq. (4) will cause the free electrons spin precession during the interaction with the optical lattice, so that the electron diffraction patterns of the two spin directions are coupled (see Figure S7 in the Supplementary Materials [40]).

**Table 1:** Theoretical results of laser wavelength and intensity for obtaining spin-dependent KD effect. With 2D optical lattice, the available visible-infrared laser beam with greatly reduced intensity could generate spin-dependent KD effect.

REF	Dimension	Laser wavelength (nm)	Laser intensity (W/cm^2^)
[32]	1D standing wave	0.4	2.0 × 10^23^
[34]	1D standing wave	0.159	1.1 × 10^22^
[35]	1D standing wave	6.2	2.3 × 10^20^
[36]	1D standing wave	0.159	1.1 × 10^22^
This work	2D optical lattice	400	∼10^14^
		800	∼10^13^

As mentioned in Table 1, this paper presents several intensity cases for 400 nm and *I* = ∼10^13^ W/cm^2^ for 800 nm to show that both visible and near-IR laser beam could be adopted to experimental observe the spin-dependent KD effect (see Figures S3 and S4 in Supplementary Materials [40]). The selection of the available laser system should consider the following factors. (1) It is better to use a shorter laser wavelength in consideration of the electron beam divergence angle. The optical lattice created by 400 nm laser instead of 800 nm can endure more divergence angle of electron beam. In other words, for the same electron beam, the overlap between different diffracted spots should be much less and diffraction pattern is easier to be identified for shorter wavelength. (2) Appropriate laser intensity should be determined. The laser intensities for *λ* = 400 nm and 800 nm in Table 1 are chosen to compare the different order of magnitude of the intensities. With the intensity of these given orders, the KD effect remains in Raman–Nath regime and the spin-dependent effect could be obtained. According to the relation between the ponderomotive potentials and the electron recoil kinetic energy in Eq. (4), the required laser intensities for longer wavelength are much lower than those for short wavelength (see Section II in Supplementary Materials [40]). Actually, the spin-dependent effect fades out with the decreasing intensity gradually. With longer interaction time and higher detection sensitivity, the spin-dependent diffraction patterns may also be captured with lower laser intensity according to Figure S4 in Supplementary Materials [40]. But if the laser intensity is too weak to satisfy the Raman–Nath conditions, the diffraction pattern will mostly gather in the central spot and the spin-dependent effect will not be observed. On the other hand, if the laser intensity is too high to have the ponderomotive potential energy comparable to a photon energy, the electron diffraction pattern would shift to higher-order diffraction peaks in a very short time, resulting in low probabilities of each diffraction order. This brings higher requirements to the stability and consistency of the laser system and the noise level of electron detection. (3) Besides, the laser spatial and temporal width used to construct the optical lattice should be uniform within the electron beam interaction range. On the basis of the above considerations, the wavelength and the intensity of the laser system proposed in this paper can be realized by the current technology. The high-intensity 800 nm or 400 nm lasers can be achieved by high-energy Ti:Sapphire laser system [41], [42] with pulse duration stretching and optional frequency doubling devices [43].

In addition, the electron pulses used to verify the spin-dependent KD effect should be well-collimated and carry the same spin. This would ensure that the diffraction pattern with obvious broken spatial inversion symmetry can be generated after electrons passing through the optical lattice. Such spin-polarized electron pulses could be realized by applying circularly polarized laser on negative-affinity photocathodes [44], [45], [46] according to the spin selection rule.

## 4 Conclusions

In conclusion, we propose a method to achieve spin-dependent KD effect by applying 2D triangular optical lattice, which is created by three linearly polarized laser beams. The spin-dependent diffraction patterns with broken spatial inversion symmetry could be obtained with the wavelength relaxed from X-ray to visible or near-infrared laser and the laser intensity is lowered by nearly five orders of magnitude. The greatly relaxed condition is owing to the relatively enhanced spin-dependent potential and weakened spin-independent potential by carefully adjusting the laser polarization. This work provides the feasibility of realizing spin-dependent KD effect based on the commercially available laser and devices. It is expected to achieve coherent and lossless manipulation of the spin states of electron beams, such as structured electron beams analogous to structured light [47], [48], quantum random walk [49] based on spin-dependent electron, and even the century-old problem of realizing the Stern–Gerlach effect with free electrons [50]. Furthermore, the spin-dependent KD effect can inspire profound exploration of spin effects in the interaction between light and magnetic or chiral samples [51], [52], as well as abundant ultrafast dynamics [53] with ultrafast electron microscope.

## Supplementary Material

Supplementary Material Details
