# Supplementary material for: Free electrons spin-dependent Kapitza–Dirac effect in two-dimensional triangular optical lattice
Source: Nanophotonics. 2024 Jul 12;13(20):3825–33. doi: 10.1515/nanoph-2024-0191 (PMC11465987; doi:10.1515/nanoph-2024-0191)
Supplement: Supplementary file 1 — Supplementary Material Details [file j_nanoph-2024-0191_suppl_001.docx]

Supplemental material for :

Free Electrons Spin-dependent Kapitza-Dirac Effect in Two-dimensional Triangular Optical Lattice

**I. Derivation of the time-independent potential function of free electron in the optical lattice**

The weak relativistic Dirac equation is introduced by using the Foldy-Wouthuysen transformation [1],

$$i\hbar\frac{\partial}{\partial t}\psi=(\frac{\left[ -i\hbar\nabla-q\vec{A} \right]^{2}}{2m}-\frac{q\hbar}{2m}\vec{\sigma}\cdot\vec{B}\boldsymbol{-}\frac{\left[ -i\hbar\nabla-q\vec{A} \right]^{4}}{8m^{3}c^{2}}-\frac{q\hbar}{4m^{2}c^{2}}\vec{\sigma}\boldsymbol{\cdot}\left\{ \vec{E}\boldsymbol{\times}\left( \boldsymbol{-}i\hbar\nabla\boldsymbol{-}q\vec{A} \right) \right\}$$

$$+\frac{q\hbar}{8m^{3}c^{2}}\{\vec{\sigma}\cdot\vec{B}\boldsymbol{,}\left[ -i\hbar\nabla-q\vec{A} \right]^{2}\})\psi(S1)$$

where $\psi$ is Pauli spinor, *m* is electron mass, *q* is electron charge, $\vec{E}$ and $\vec{B}$ are the electric and the magnetic field, $\vec{A}$ is the vector potential and $\vec{\sigma}\boldsymbol{=(}\sigma_{x},\sigma_{y},\sigma_{z}\boldsymbol{)}$ is the Pauli matrices. The first term and the second term on the right side are consistent with the Schrödinger equation, while the third term is the relativistic correction to the first term. The fourth term on the right side leads to the spin-orbit coupling and coupling of the optical lattice pseudospin to the electron spin. The last term is anticommutator, representing the correction to the Zeeman coupling [33]. When the polarization angle is $\theta$, the vector potential of the three laser beams can be written as,

$$\begin{aligned} \vec{A}_{1}=\frac{E_{0}}{\omega}\sin\left( \vec{k}_{1}\cdot\vec{r}-\omega t \right)\left( \vec{e}_{1}\sin\left( \theta\right)+\hat{n}\cos\left( \theta\right) \right) \end{aligned}$$

$$\begin{aligned} \vec{A}_{2}=\frac{E_{0}}{\omega}\sin\left( \vec{k}_{2}\cdot\vec{r}-\omega t \right)\left( \vec{e}_{2}\sin\left( \theta\right)+\hat{n}\cos\left( \theta\right) \right)\#\left( S2 \right) \end{aligned}$$

$$\vec{A}_{3}=\frac{E_{0}}{\omega}\sin\left( \vec{k}_{3}\cdot\vec{r}-\omega t \right)\left( \vec{e}_{3}\sin\left( \theta\right)+\hat{n}\cos\left( \theta\right) \right)$$

where $\vec{e}_{i}=\hat{k}_{i}\times\hat{n}$ and $\hat{n}$ is the unit vectors, representing the parallel and the perpendicular components of $\vec{A}_{i}$ regarding to the optical lattice plane, respectively. Under the conditions of the laser wavelength and the intensity assumed in this work, Eq. S1 could be simplified to the following form by comparing the coefficients of each term in the Hamiltonian and neglecting the high-order small terms,

$$\begin{aligned} i\hbar\frac{\partial}{\partial t}\psi=\left( \frac{{(-i\hbar\nabla-q\vec{A})}^{2}}{2m}-\frac{q\hbar}{2m}\vec{\sigma}\cdot\vec{B}+\frac{q\hbar}{4m^{2}c^{2}}\vec{\sigma}\boldsymbol{\cdot(}\vec{E}\boldsymbol{\times}q\vec{A}) \right)\psi\#\left( S3 \right) \end{aligned}$$

where the neglected terms are at least two orders of magnitude smaller than the reserved terms. According to the Magnus expansion, the time evolution of the wave function can be expressed as [2,3],

$$\begin{aligned} \psi\left( t \right)=\exp\left( \tilde{U}_{1}\left( t \right)+\tilde{U}_{2}\left( t \right)+\ldots\right)\psi\left( 0 \right)\#\left( S4a \right) \end{aligned}$$

$$\begin{aligned} \tilde{U}_{1}\left( t \right)=\frac{1}{i\hbar}\int_{0}^{t} dt_{1}H\left( t_{1} \right)\#\left( S4b \right) \end{aligned}$$

$$\begin{aligned} \tilde{U}_{2}\left( t \right)=\frac{1}{2}\left( \frac{1}{i\hbar} \right)^{2}\int_{0}^{t} dt_{1}\int_{0}^{t_{1}} dt_{2}\left[ H\left( t_{1} \right),H\left( t_{2} \right) \right]\#\left( S4c \right) \end{aligned}$$

$$\begin{aligned} \tilde{U}_{3}\left( t \right)=\frac{1}{6}\left( \frac{1}{i\hbar} \right)^{3}\int_{0}^{t} dt_{1}\int_{0}^{t_{1}} dt_{2}\int_{0}^{t_{2}} dt_{3}\left( \left[ H\left( t_{1} \right),\left[ H\left( t_{2} \right),H\left( t_{3} \right) \right] \right]+\left[ \left[ H\left( t_{1} \right),H\left( t_{2} \right) \right],H\left( t_{3} \right) \right] \right)\#\left( S4d \right) \end{aligned}$$

where the square brackets calculate the commutator of the two Hamiltonians inside. The first and the third term on the right part of Eq. S3 contribute to the first order Magnus expansion shown as Eq. S4b. Extracting the terms that grow linearly with time leads to

$$\begin{aligned} \tilde{U}_{1}\left( t \right)=\frac{1}{i\hbar}\int_{0}^{t} dt_{1}H\left( t_{1} \right)=\frac{1}{i\hbar}t\left( -\frac{\hbar^{2}}{2m}\nabla^{2}+H_{A^{2}}+H_{E\times A} \right)\#\left( S5 \right) \end{aligned}$$

$$\begin{aligned} H_{A^{2}}=\frac{q^{2}}{2m}\frac{{E_{0}}^{2}}{\omega^{2}}\left( \frac{3}{2}+\left( -\frac{1}{2}\sin^{2} \left( \theta\right)+\cos^{2} \left( \theta\right) \right)\left( \cos\left( \vec{Q}_{1}\cdot\vec{r} \right)+\cos\left( \vec{Q}_{2}\cdot\vec{r} \right)+\cos\left( \vec{Q}_{3}\cdot\vec{r} \right) \right) \right)\#\left( S5a \right) \end{aligned}$$

$$\begin{aligned} H_{E\times A}=\frac{q^{2}\hbar}{4m^{2}c^{2}}\frac{E_{0}^{2}}{\omega}\sin\left( \frac{2\pi}{3} \right)\vec{\sigma}\cdot\left\{ \begin{aligned} \left( \sin\left( \vec{Q}_{1}\cdot\vec{r} \right)+\sin\left( \vec{Q}_{2}\cdot\vec{r} \right)+\sin\left( \vec{Q}_{3}\cdot\vec{r} \right) \right)\hat{z}\sin^{2} \left( \theta\right) \\ +\sin\left( \vec{Q}_{1}\cdot\vec{r} \right)\hat{Q}_{1}\sin\left( 2\theta\right) \\ +\sin\left( \vec{Q}_{2}\cdot\vec{r} \right)\hat{Q}_{2}\sin\left( 2\theta\right) \\ +\sin\left( \vec{Q}_{3}\cdot\vec{r} \right)\hat{Q}_{3}\sin\left( 2\theta\right) \end{aligned} \right\}\#\left( S5b \right) \end{aligned}$$

where $\vec{Q}_{i}\in\left\{ \vec{Q}_{l}{=\vec{k}}_{i}-\vec{k}_{j} | \left( i, j, l \right)\in\left\{ \left( 3,2,1 \right), \left( 1,3,2 \right), \left( 2,1,3 \right) \right\} \right\}$, $\hat{Q}_{i}$ is the direction vector of $\vec{Q}_{i}$, and $E_{0}$ is the electric field amplitude. Obviously the first order Magnus expansion already contains the spin-dependent potential indicated by the Pauli matrices in Eq. S5b. Moreover, the second term on the right part of Eq. S3 contributes to the second order Magnus expansion,

$$\begin{aligned} \tilde{U}_{2}\left( t \right)=\frac{1}{2}\left( \frac{1}{i\hbar} \right)^{2}\int_{0}^{t} dt_{1}\int_{0}^{t_{1}} dt_{2}\left[ H\left( t_{1} \right),H\left( t_{2} \right) \right] \\ =\frac{1}{2}\left( \frac{1}{i\hbar} \right)^{2}\int_{0}^{t} dt_{1}\int_{0}^{t_{1}} dt_{2}\frac{q^{2}\hbar^{2}}{4m^{2}}\left( \vec{\sigma}\cdot\vec{B}\left( t_{1} \right)\vec{\sigma}\cdot\vec{B}\left( t_{2} \right)-\vec{\sigma}\cdot\vec{B}\left( t_{2} \right)\vec{\sigma}\cdot\vec{B}\left( t_{1} \right) \right)\#\left( S6 \right) \end{aligned}$$

To simplified the expression, we use the commutation relation between Pauli matrices,

$$\begin{aligned} \left[ \sigma_{i},\sigma_{j} \right]=2i\varepsilon_{ijk}\sigma_{k}\#\left( S7 \right) \end{aligned}$$

where $\varepsilon_{ijk}$ is the Levi-Civita symbol. The right part of Eq. S7 represents Einstein summation notation. With the commutation relation the second order expansion can be written in a quite simple way as follows,

$$\begin{aligned} \tilde{U}_{2}\left( t \right)=\frac{t}{i\hbar}\frac{q^{2}\hbar}{4m^{2}}\frac{{E_{0}}^{2}}{{\omega c}^{2}}\sin\left( \frac{2\pi}{3} \right)\vec{\sigma}\cdot\left\{ \begin{aligned} -\left( \sin\left( \vec{Q}_{1}\cdot\vec{r} \right)+\sin\left( \vec{Q}_{2}\cdot\vec{r} \right)+\sin\left( \vec{Q}_{3}\cdot\vec{r} \right) \right)\hat{z}\cos^{2} \left( \theta\right) \\ +\sin\left( \vec{Q}_{1}\cdot\vec{r} \right)\hat{Q}_{1}\sin\left( 2\theta\right) \\ +\sin\left( \vec{Q}_{2}\cdot\vec{r} \right)\hat{Q}_{2}\sin\left( 2\theta\right) \\ +\sin\left( \vec{Q}_{3}\cdot\vec{r} \right)\hat{Q}_{3}\sin\left( 2\theta\right) \end{aligned} \right\}\#\left( S6 \right) \end{aligned}$$

Since the Pauli matrices do not commutate with each other, the $\vec{\sigma}\cdot\vec{B}$ term would contribute to the third Magnus expansion in Eq. S4d. We find that the third Magnus expansion term is nearly two orders of magnitude smaller than the second expansion term. Thus, the third and higher order expansion terms can be ignored.

In the Magnus expansion, we have ignored the role of the terms linearly related to the vector potential which oscillate fast at laser frequency. This is because our diffraction pattern results are sampled at the interaction time of integer laser periods. In the Magnus expansion, at the interaction time of integer laser periods, the oscillating terms tend to zero in the integral over time [4]. However, at interaction time of non-integer periods, those terms linearly related to field components could have great influence on the diffraction results. Due to stimulated Compton scattering, the electrons will only occupy *m*$\vec{b}_{1}$+*n*$\vec{b}_{2}$ (*m, n*$\mathbb{\in Z}$) in the momentum space. According to the interference between different paths, electrons may appear on *p*$\vec{k}_{1}$+*s*$\vec{k}_{2}$ (*p, s*$\mathbb{\in Z}$) and may even cover the influence of the ponderomotive potentials, making it difficult for us to observe spin-dependent diffraction patterns at interaction time of non-integer laser periods.

Under practical conditions, the changes of diffraction patterns caused by the linear terms are neglected by integral over time, which depends on the interaction time and the temporal shape of laser pulse, while the effect of the ponderomotive potential terms would grow continuously during the interaction. For instance, on the time scale considered in this article, which includes thousands of laser cycles under the Gaussian envelope, the integral of the linear terms over time is quite small, making the effect of the ponderomotive potential terms dominant.

The energy change of electrons comes from both the linear terms of field and the ponderomotive potential terms in the interaction. The effect of the ponderomotive potential terms on the velocity is negligible, because the KD effect is actually the stimulated Compton scattering where the two involving photons have the same energy. Therefore, the velocity of the electron only changes direction slightly during the scattering process, forming the diffraction patterns. The energy change resulting from the linear terms of field is similar to the diffraction discussed above, so the energy change tends to zero when the interaction time contains integer laser periods. Besides, with the temporal envelope of the laser changing much more slowly than the laser oscillation periods, the energy change of the electron after interacting with the optical lattice is thus very small according to the integral over time.

**II. The relations between the ponderomotive potentials and wavelength, polarization direction and laser intensity**

The relative amplitude ratio of the spin-dependent potential $V_{spin}$and the spin-independent potential $V_{0}$ decides whether the feature of spatial inversion symmetry breaking diffraction pattern is obvious or not. As indicated by Eq. 4 in the main text, both the $V_{spin}$ and $V_{0}$ are influenced by the laser intensity, wavelength and polarization direction angle $\theta$.


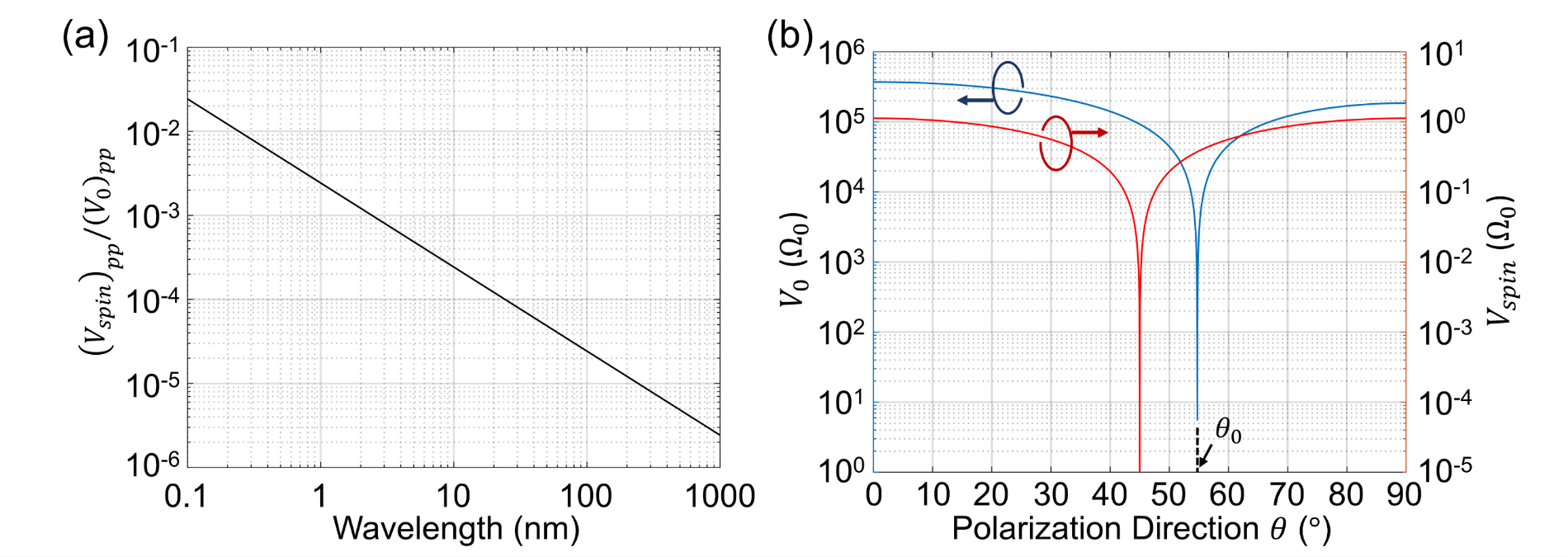


Fig. S1. The dependence of ponderomotive potentials on the laser wavelength and polarization direction. (a) The relative amplitude ratio $\left( V_{spin} \right)_{pp}/\left( V_{0} \right)_{pp}$ is plotted with wavelength varying from 0.1nm to 1000nm when the polarization is parallel to the optical lattice. The straight line indicates the inversely proportion to the wavelength. (b) The ponderomotive potentials as a function of polarization angle *θ* with wavelength fixed as $\lambda$ = 400nm. $V_{0}$ can tend to zero when polarization angle is $\theta_{0}$. The unit of ponderomotive potentials is $\Omega_{0}={(qE_{0}\hbar c)}^{2}/\hbar\omega{(mc^{2})}^{2}$.

The spatial inversion symmetry properties of $V_{spin}$ and $V_{0}$ are quite different. Since $V_{0}\propto{E_{0}}^{2}/\omega^{2}$ and $V_{spin}\propto{E_{0}}^{2}/\omega$, their amplitude ratio $\left( V_{spin} \right)_{pp}/\left( V_{0} \right)_{pp}$ is predominated by the laser wavelength when the laser polarization direction is fixed as shown by Eq. 5 in the main text. The relation between $\left( V_{spin} \right)_{pp}/\left( V_{0} \right)_{pp}$ and the laser wavelength is illustrated in Fig. S1(a) with *θ* = $\pi/2$. The straight and decreasing line in the double-logarithm plot indicates the extremely weak spin-dependent effect in the visible to near-infrared band, while spin-dependent effect may arise in X-ray or EUV band.

In addition to the laser wavelength, the polarization direction of laser beam also makes a great contribution to realize the final results in our work. The variation of $V_{\mathrm{spin}}$and $V_{0}$ with the polarization direction angle *θ* is illustrated individually in Fig. S1(b), as an additional aspect for Fig. 2 in the main text. The laser wavelength is 400nm. The general scale of ponderomotive potentials $\Omega_{0}={(qE_{0}\hbar c)}^{2}/\hbar\omega{(mc^{2})}^{2}$ is introduced for eliminating the influence from the laser intensity. When the polarization is adjusted from perpendicular to parallel to the optical lattice, the $V_{\mathrm{spin}}$ turns to zero near *θ*=45° shown as the red line. Then, the $V_{0}$ is highly suppressed near $\theta_{0}$ ($arcsin(\sqrt{2/3})$), shown as the blue line, where we carry out the spin-dependent KD effect calculation with laser wavelength $\lambda$ = 400nm. Otherwise, the $V_{0}$ is several orders of magnitude stronger than the $V_{\mathrm{spin}}$, inducing no obvious spin-dependent effect in the interaction.


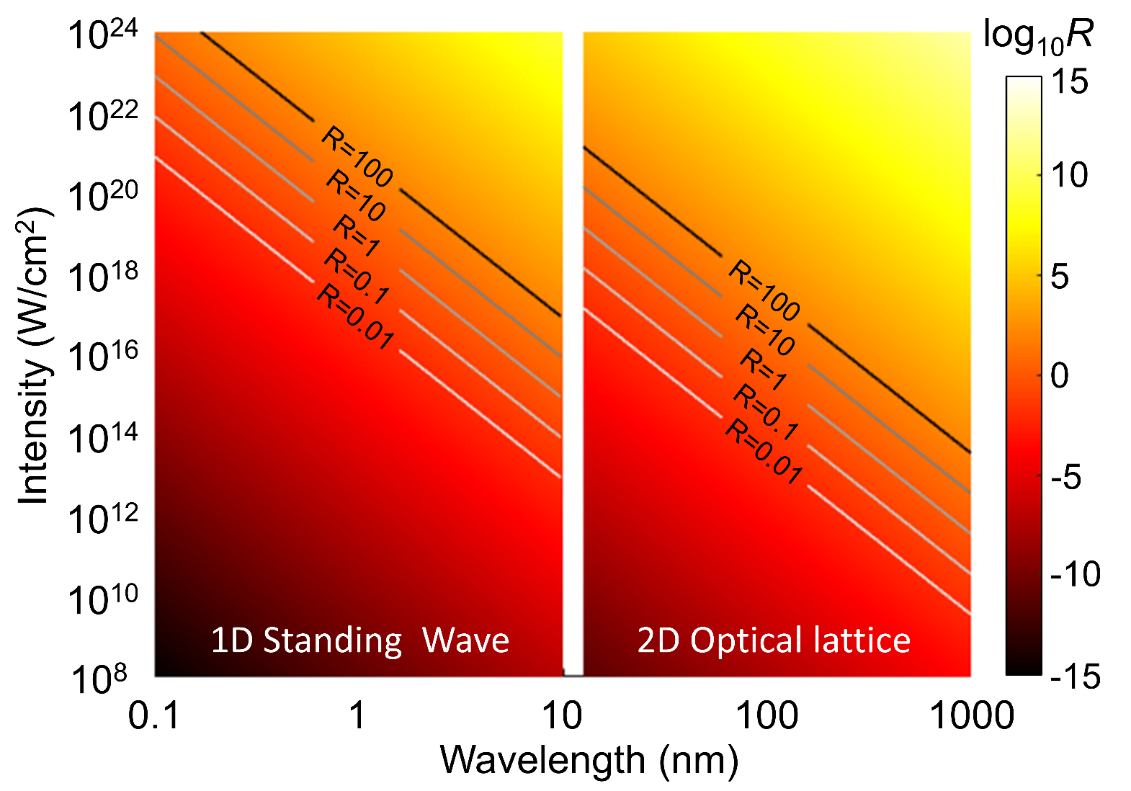


Fig. S2. The ratio *R* of spin-independent ponderomotive potential and recoil kinetic energy versus wavelength and intensity. The left and the right parts represent 1D standing wave with short wavelength and 2D optical lattice with longer wavelength, respectively. The contours where R=0.01, 0.1, 1, 10 and 100 are plotted. The polarization angle is $\theta=\text{54.735°}$ in the right part calculation.

The importance of the laser intensity for spin-dependent KD effect has been discussed in the main text. Whether in the short wavelength 1D standing wave or in the long wavelength 2D optical lattice discussed in this paper, the spin-independent ponderomotive potential is much larger than the spin-dependent one. To show neatly why the required intensity drops by several orders of magnitude for visible or near-infrared optical lattice compared with the previously reported short wavelength cases, we define *R* as the ratio of the spin-independent ponderomotive potential to the electron recoil kinetic energy, which is illustrated in Fig. S2.

The contours of different *R* have been outlined. $R\gg1$ indicates the Raman-Nath regime, which is the situation in this paper. To meet this condition, the required laser intensity is much lower for longer wavelength. On the other hand, $R\sim1$ is usually chosen in previous works in order to avoid too weak interaction [5,6], which results in the required intensity being higher than 10^20^W/cm^2^. Indeed, the much lower intensities given in Table 1 in the main text is confirmed by Fig. S2.

**III. Other supplemental figures.**

In this section the diffraction patterns in the visible or near-IR optical lattice is calculated with polarization angle $\theta=\text{54.735°}$ as in the main text.

Fig. S3. The spin-dependent KD effect in the X-ray and near-IR optical lattice.

Fig. S4. The spin-dependent KD effect in the 400nm optical lattice with different laser intensity.

Fig. S5. The spatial inversion symmetry breaking in higher-order diffraction spots.

Fig. S6. Diffraction patterns of spin $+\hat{z}$ free electrons with different interaction time.

Fig. S7. The spin-flipping of free electron with initial spin $+\hat{z}$ in triangular optical lattice due to spin precession potential $V_{p}$.


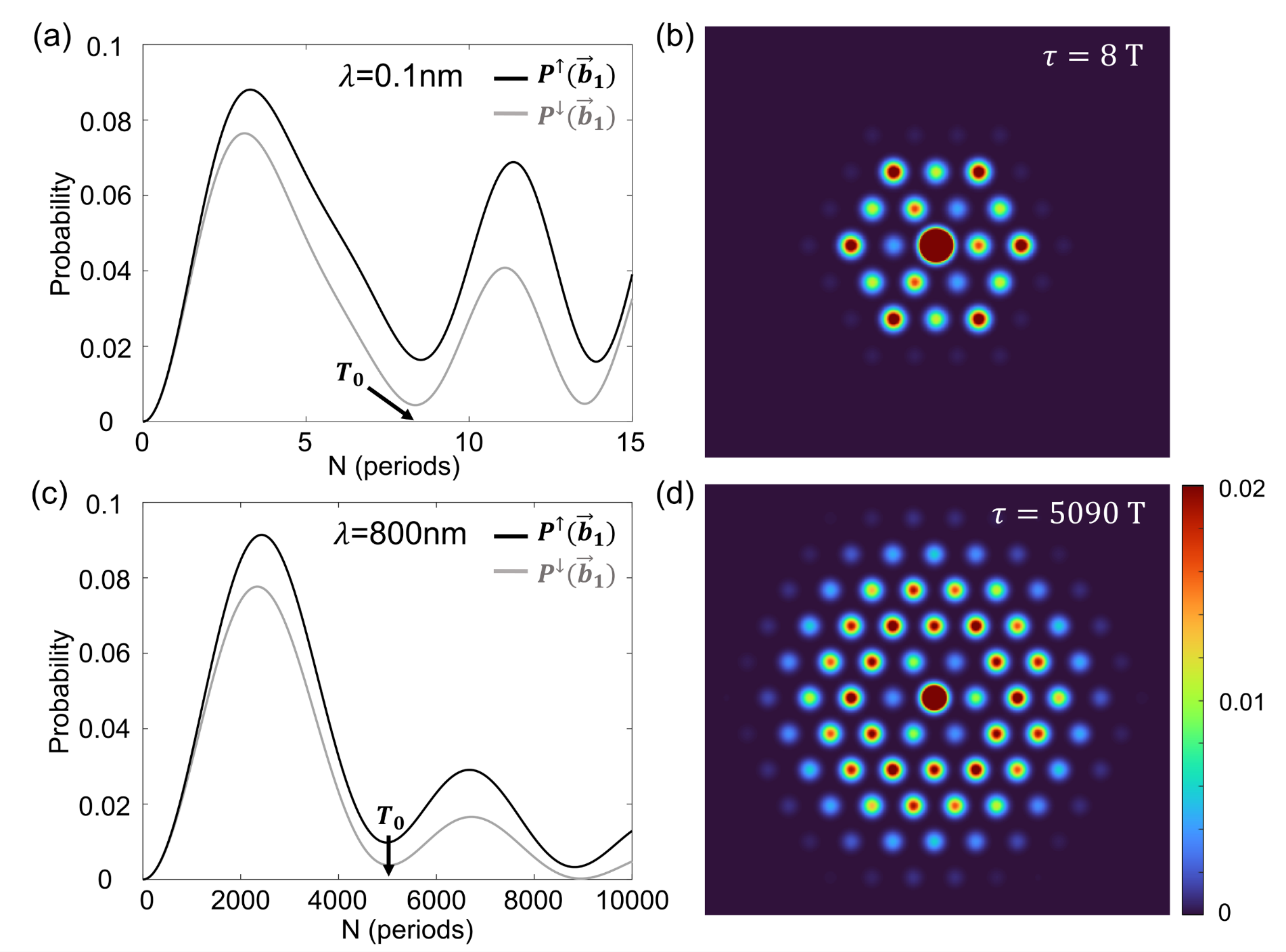


Fig. S3. The evolution of electron diffraction probabilities and the diffraction patterns in the X-ray and near-IR optical lattice. (a) The optical lattice is created with X-ray of $\lambda$ = 0.1nm and *E* = 2.2×10^15^*V*/*m* (corresponding to the intensity 6.4×10^23^W/cm^2^). In this case the polarization of lasers is parallel to the optical lattice plane, *θ*=90°. The black and the grey line represent the probability at $\vec{b}_{1}$ spot of the spin direction $+\hat{z}$ and $-\hat{z}$, that is $P^{\uparrow}(\vec{b}_{1})$ and $P^{\downarrow}(\vec{b}_{1})$, respectively. The potential of optical lattice experienced by spin $+\hat{z}$ electrons is shown in Fig. 2(b) in the main text. (b) The diffraction pattern of spin $+\hat{z}$ electrons, sampled at interaction time $\tau$ = 8T (T is the laser oscillation period). The quite short interaction time results from the extremely high intensity in Raman-Nath regime under X-ray wavelength. (c) The optical lattice is created with infrared lasers of $\lambda$ = 800nm and *E* = 2×10^10^*V*/*m* (corresponding to the intensity 5.3×10^13^W/cm^2^). (d) The diffraction pattern of spin $+\hat{z}$ electrons, sampled at interaction time $\tau$ = 5090T (~13.6ps). Spatial inversion symmetry breaking and C3-symmetry property can be identified both in (b) and (d), showing that the spin-dependent KD effect could also come up in X-ray and infrared 2D optical lattice. $T_{0}$ in the figures represents when $P^{\uparrow}(\vec{b}_{1})$ and $P^{\downarrow}(\vec{b}_{1})$ reach a local minimum.


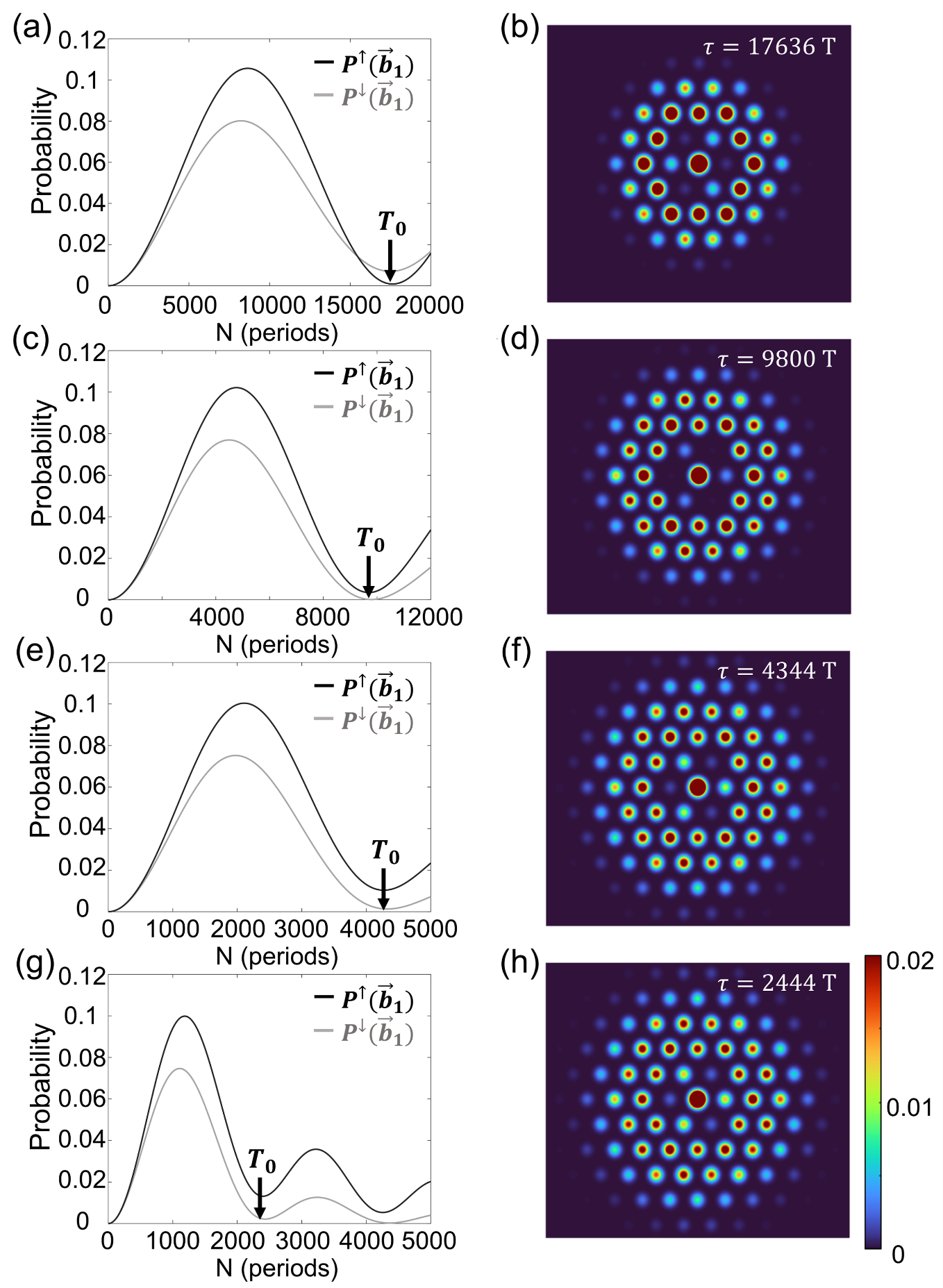


Fig. S4. The evolution of electron diffraction probabilities and the diffraction patterns under the same wavelength $\lambda$= 400nm but different laser intensity. (a), (c), (e) and (g) are calculated with *E* = 3.0×10^10^*V*/*m*, 4.0×10^10^*V*/*m*, 6.0×10^10^*V*/*m* and 8.0×10^10^*V*/*m*, respectively. The black line and the grey line represent the probability at $\vec{b}_{1}$ spot of the spin direction $+\hat{z}$ and $-\hat{z}$. (b), (d), (f) and (h) are the diffraction patterns of spin $+\hat{z}$ electrons under the conditions corresponding to (a), (c), (e) and (g). The patterns are sampled at the interaction time when $P^{\uparrow}(\vec{b}_{1})$ reaches the first minimum, that is (b) $\tau=$17636T (~23.5ps), (d) $\tau=$9800T (~13.7ps), (f) $\tau=$4344T (~5.8ps) and (h) $\tau=$2444T (~3.3ps). Spatial inversion symmetry breaking and C3-symmetry property can be identified in all the diffraction patterns. The results also show that the diffraction probabilities oscillate much faster as the laser intensity increases. Notice that $-\vec{b}_{1}$ spot is brighter than $\vec{b}_{1}$ spot in (b), indicating a higher diffraction probability at $-\vec{b}_{1}$ spot. While in (d), (f) and (h), the $-\vec{b}_{1}$ spot is darker than $\vec{b}_{1}$ spot. This is because the laser intensity in (a) and (b) is relatively weak and keeps the electron-optical lattice scattering away from the Raman-Nath regime. As a result, the electron diffraction in (a) and (b) is limited in few orders, leading to the “reflection” from the higher-order to lower-order spots, causing the unusual diffraction pattern. $T_{0}$ in the figures represents when $P^{\uparrow}(\vec{b}_{1})$ and $P^{\downarrow}(\vec{b}_{1})$ reach a local minimum.


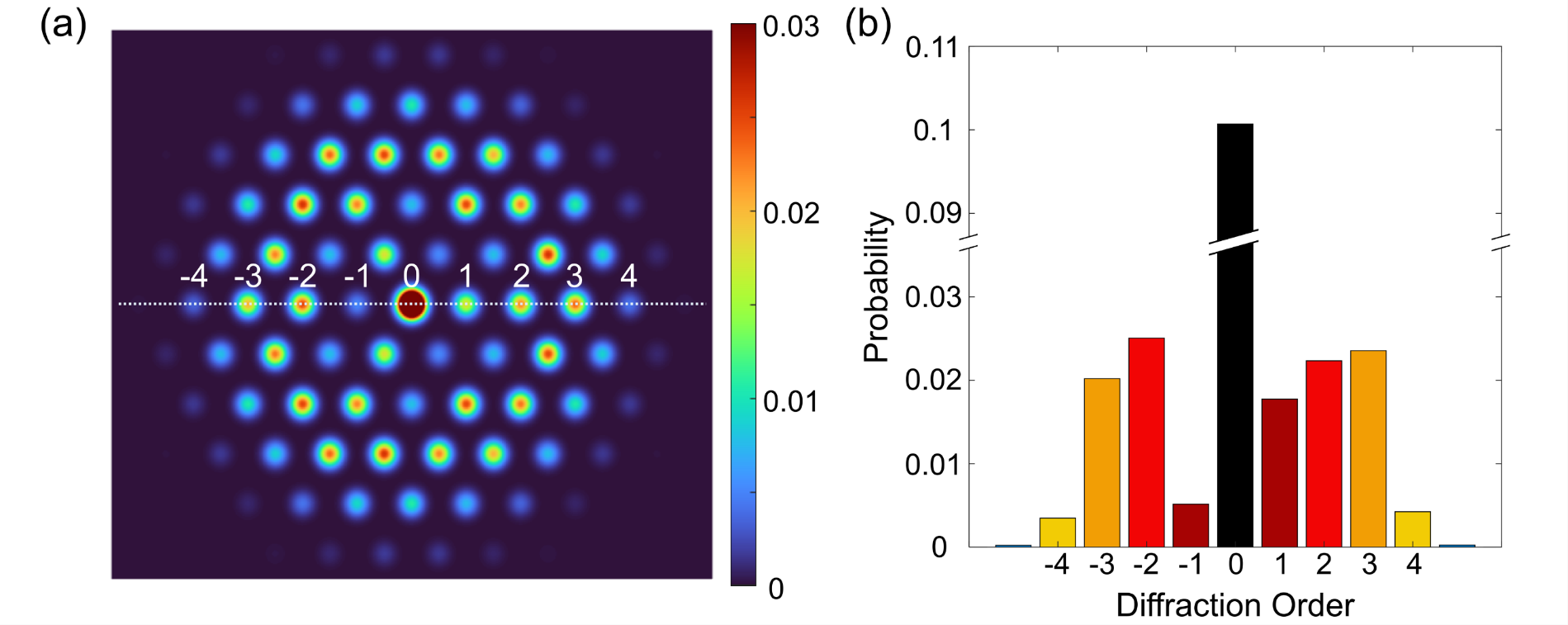


Fig. S5. Diffraction pattern and probabilities of spin $+\hat{z}$ electrons at higher-order diffraction spots. (a) Free electron diffraction pattern at interaction time $\tau=$ 7000T (~9.3ps). The probabilities of (-4^th^~+4^th^)-order spots on the white dashed line are illustrated in (b). Just like the different probabilities of 1^st^-order spots, the asymmetry of 2^nd^~4^th^-order spots indicates the spatial inversion symmetry breaking, despite the low visibilities or the low probabilities. Here the optical lattice is formed with $\lambda$= 400nm and *E* = 5.0×10^10^*V*/*m* laser as in the main text.


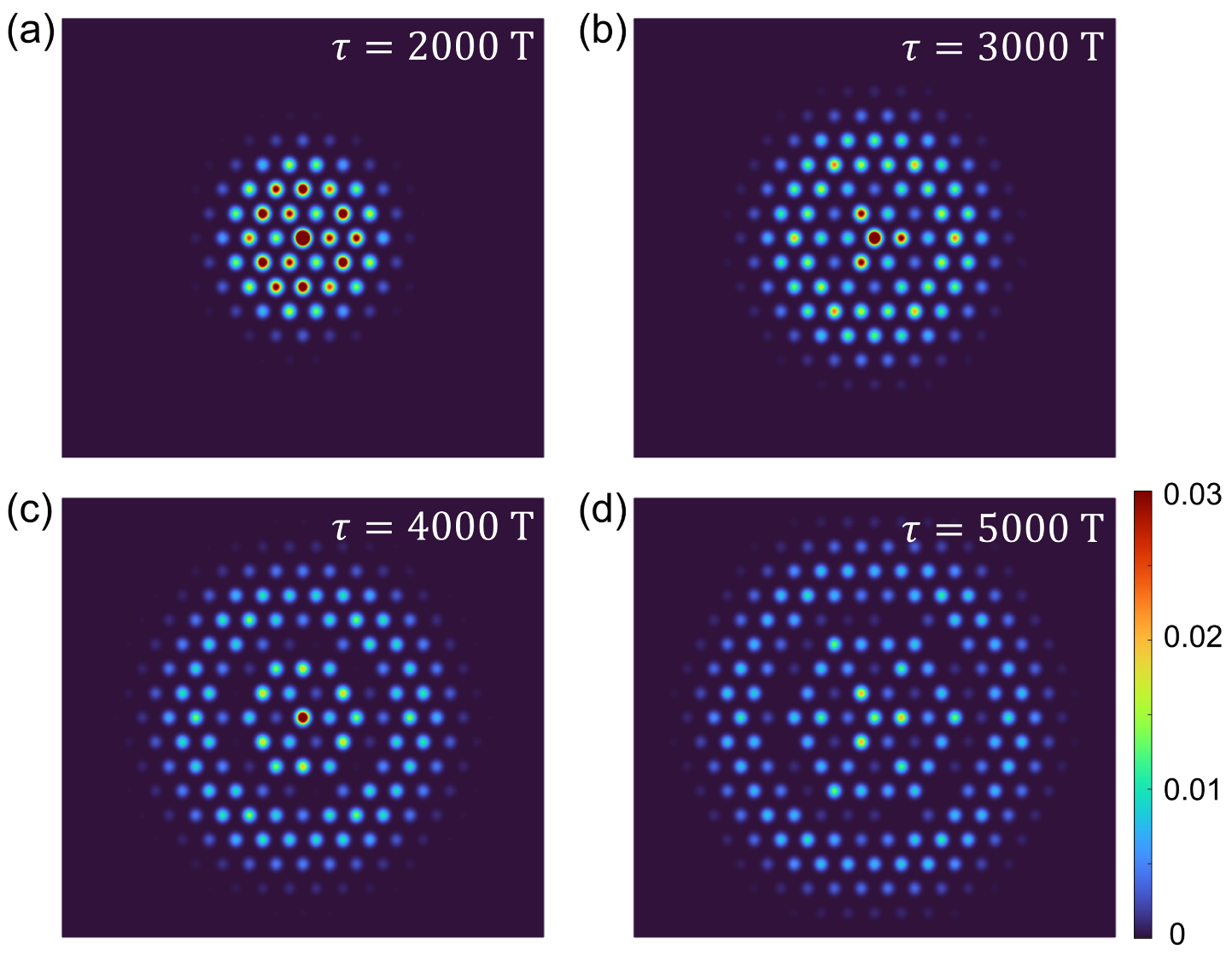


Fig. S6. Diffraction patterns of spin $+\hat{z}$ free electrons with the interaction time $\tau=$ (a) 2000T, (b) 3000T, (c) 4000T and (d) 5000T. In this case the optical lattice is formed with $\lambda$ = 400nm laser as in the main text but the electric field is enhanced to *E* = 8.0×10^10^*V*/*m*. The 1^st^-order diffraction spots show apparent spatial inversion symmetry breaking and C3-symmetry in all the diffraction patterns. As the interaction time is extended, the higher-order diffraction spots become more obvious. Only 1^st^~4^th^-order diffraction spots can be identified in (a), while the 1^st^~8^th^-order diffraction spots appear obviously after 3000 laser periods in (d).


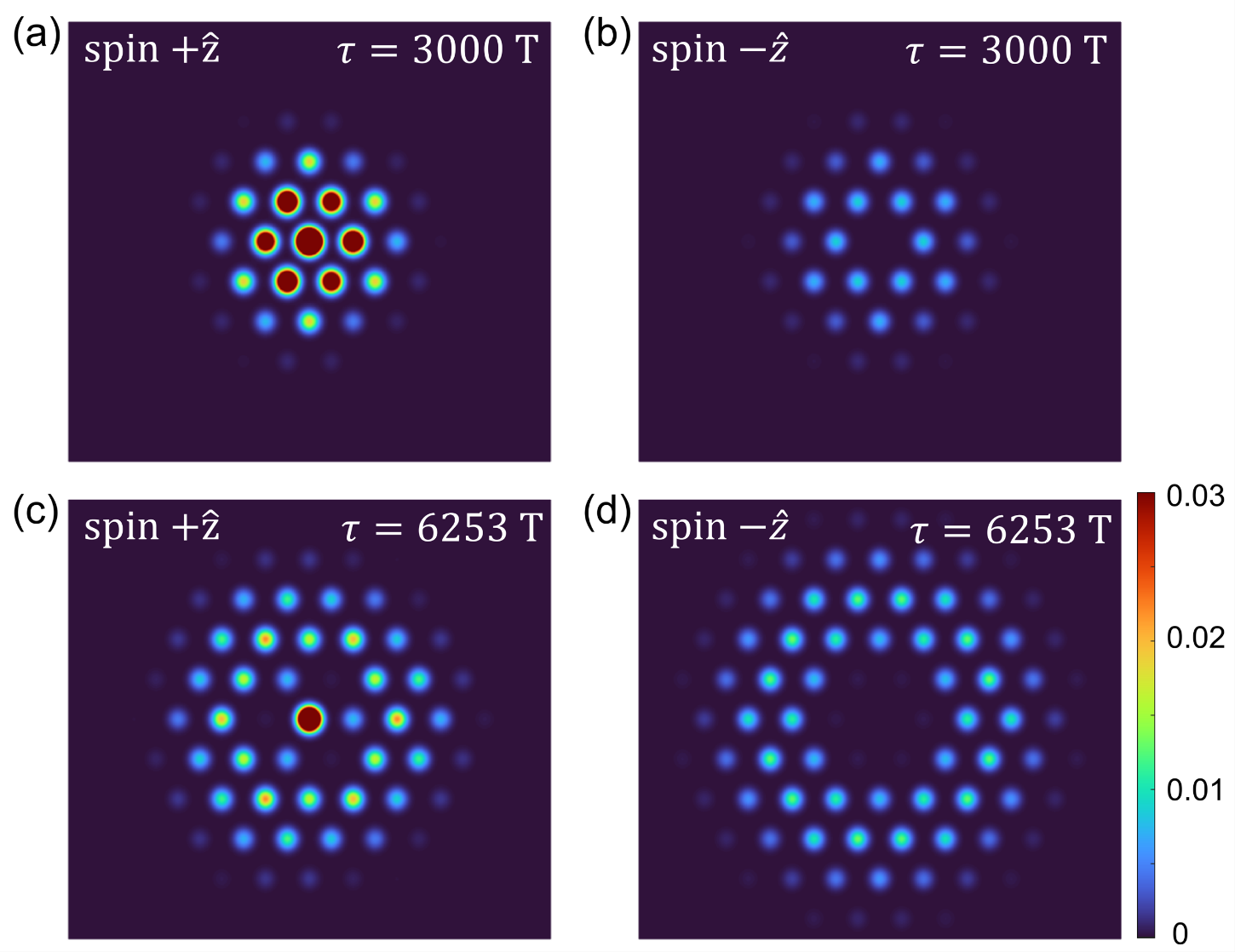


Fig. S7. The spin-flipping of free electron with initial spin $+\hat{z}$ in triangular optical lattice due to spin precession potential $V_{p}$. (a)(b) The diffraction patterns for spin $+\hat{z}$ and spin $-\hat{z}$ free electron with the interaction time $\tau=$3000T. Even though the free electron has initial spin $+\hat{z}$, the spin $-\hat{z}$ electrons come up because of the in-plane Pauli matrices in spin precession potential $V_{p}$. The spin-flipping is accompanied by momentum transfer, that is, the first order diffraction spots in (b) mainly come from the central spot in (a). The spin $-\hat{z}$ electron has very low probability with short interaction time and it becomes apparent when extending the interaction time as (c)(d), where the spin $-\hat{z}$ electron locates in high order diffraction spots and only has little impact on the spatial inversion symmetry breaking property in the first order spots.

Reference

[1] L. L. Foldy and S. A. Wouthuysen, "On the Dirac theory of spin 1/2 particles and its non-relativistic limit," *Physical Review*, vol. 78, no. 1, pp. 29-36, 1950, https://doi.org/10.1103/PhysRev.78.29.

[2] W. Magnus, "On the exponential solution of differential equations for a linear operator," Comm. Pure Appl. Math. vol. 7, 1954, https://doi.org/10.1002/cpa.3160070404.

[3] S. Blanes, F. Casas, J. A. Oteo, and J. Ros, "The Magnus expansion and some of its applications," *Physics reports*, vol. 470, no. 5, pp. 151-238, 2009, https://doi.org/10.1016/j.physrep.2008.11.001.

[4] N. Talebi and C. Lienau, "Interference between quantum paths in coherent Kapitza–Dirac effect," New Journal of Physics, vol. 21, no. 9, p. 093016, 2019, https://doi.org/10.1088/1367-2630/ab3ce3.

[5] R. Erhard and H. Bauke, "Spin effects in Kapitza-Dirac scattering at light with elliptical polarization," *Physical Review A*, vol. 92, no. 4, p. 042123, 2015, https://doi.org/10.1103/PhysRevA.92.042123.

[6] S. Ahrens, "Electron-spin filter and polarizer in a standing light wave," *Physical Review A*, vol. 96, no. 5, p. 052132, 2017, https://doi.org/10.1103/PhysRevA.96.052132.
